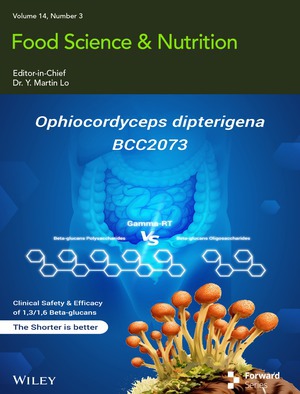# Cover Image

**DOI:** 10.1002/fsn3.71718

**Published:** 2026-05-06

**Authors:** Numphung Rungraung, Niramol Muangpracha, Pakkapong Phucharoenrak, Wai Prathumpai, Dunyaporn Trachootham

## Abstract

The cover image is based on the article *Differential Safety and Lipid Control Efficacy of β‐1,3/1,6‐Glucan Oligosaccharides and Polysaccharides Derived From Ophiocordyceps dipterigena BCC 2073 in Healthy Volunteers* by Numphung Rungraung et al., https://doi.org/10.1002/fsn3.71379.